# Clinicopathological Review of 547 Bulbar Enucleations in Hungary (2006–2017)

**DOI:** 10.1155/2019/2042459

**Published:** 2019-02-14

**Authors:** Gábor Tóth, Nóra Szentmáry, Gábor László Sándor, Béla Csákány, Erika Maka, Jeannette Tóth, Zsuzsanna Antus, Milán Tamás Pluzsik, Achim Langenbucher, Zoltán Zsolt Nagy, Olga Lukáts

**Affiliations:** ^1^Department of Ophthalmology, Semmelweis University, Budapest, Hungary; ^2^Department of Ophthalmology, Saarland University Medical Center, UKS, Homburg, Saar, Germany; ^3^2nd Department of Pathology, Semmelweis University, Budapest, Hungary; ^4^Department of Ophthalmology, Bajcsy-Zsilinszky Hospital, Budapest, Hungary; ^5^Experimental Ophthalmology, Saarland University, Homburg, Saar, Germany

## Abstract

**Purpose:**

To analyse current clinicopathological enucleation indications in a large third-referral centre in a developed country (Hungary) over a period of 12 years.

**Methods:**

Retrospective review was performed on 547 enucleated eyes of 543 patients (48.6% males, age 52.7 ± 24.5 years) who were operated on between 2006 and 2017 at the Department of Ophthalmology of Semmelweis University, in Budapest, Hungary. For each subject, clinicopathological data, including patient demographics, indications for enucleation, B-scan ultrasound reports, operative details, and histopathological analyses, were reviewed. *Primary enucleation indications* were classified into trauma, tumours, systemic diseases, surgical diseases, infections or inflammations, miscellaneous diseases, and unclassifiable groups. *Clinical immediate enucleation indications* were classified as tumours, atrophia or phthisis bulbi, infection or inflammation, painful blind eye due to glaucoma, acute trauma, threatening or spontaneous perforation, cosmetic causes, and expulsive bleeding.

**Results:**

The most common *primary enucleation indications* were tumours (47.3%), trauma (16.8%), surgical diseases (15.7%), infection or inflammation (11.6%), systemic diseases (5.1%), miscellaneous diseases (2.0%), and unclassifiable diseases (1.5%). *Clinical immediate enucleation indications* were tumours (46.1%), atrophia or phthisis bulbi (18.5%), infection or inflammation (18.5%), painful blind eye due to glaucoma (11.2%), acute trauma (3.7%), threatening or spontaneous perforation (1.3%), cosmetic reasons (0.5%), and expulsive bleeding (0.4%).

**Conclusions:**

Intraocular tumours represent the most common clinicopathological indication for ocular enucleation in our study population. Following ocular trauma and systemic diseases, the rate of enucleation decreased in the last decade, compared to those previously reported in other developed countries. However, changes were not observed for surgical diseases, infectious and inflammatory causes, or for miscellaneous and unclassified diseases. Orbital implant financing should be increased to ensure better postoperative aesthetic rehabilitation, following enucleation in Hungary.

## 1. Introduction

Enucleation is the removal of the entire globe and a section of the optic nerve. It is sometimes an unavoidable end-stage solution for several ophthalmic diseases. This procedure may be required after severe ocular trauma, tumours, infections, or painful blind eye [1]. Indications for enucleation—since the first description of the surgery in 1583 by Bartisch—may differ over time, with changing incidences of different ophthalmic conditions (e.g., diabetes mellitus, secondary glaucoma, and tumours) and therapeutic regimens (e.g., panretinal photocoagulation, intravitreal injections, and chemotherapies) [2].

There have been several clinicopathological studies on indications for enucleation, but these surveys are not current or were published in developing countries [3–7]. The order of primary clinical enucleation indications varies among different countries.

Since there is almost no up-to-date information about enucleation in European countries, the primary aim of this study was to analyse current enucleation indications in a developed country (Hungary) over a period of 12 years.

## 2. Materials and Methods

This retrospective study was undertaken at a tertiary eye care centre, to analyse the current indications for enucleation in Hungary. The study was performed in accordance with the Declaration of Helsinki Guidelines for Human Research.

This retrospective review was conducted on 547 eyes of 543 patients who underwent enucleation at the Department of Ophthalmology of Semmelweis University, between January 2006 and December 2017. For each subject, clinicopathological data were reviewed, which included patient demographics, indications for enucleation, B-scan ultrasound reports, operative details, and histopathological analyses. Final diagnosis was based on clinical history and histopathological findings. Paraffin sections stained with haematoxylin-eosin and histopathological charts were available for 535 (97.8%) globes.

*Primary enucleation indications* (classification of de Gottrau et al. [3]; based on clinical history and histological reports) were divided into seven groups: trauma, tumours (intraocular, periocular, or intraorbital), systemic diseases, surgical diseases (treated or untreated), infectious and inflammatory diseases, miscellaneous diseases, and unclassifiable diseases due to incomplete case history. Second, *immediate clinical enucleation indications* were categorised using the classification of de Gottrau et al. [3], modified with three additional diagnosis groups, such as threatening or spontaneous perforation, expulsive bleeding, and cosmetic reasons. Therefore, the immediate clinical enucleation indications were (last diagnosis before anophthalmia surgery) tumour, atrophia or phthisis bulbi, infection or inflammation, painful blind eye due to glaucoma, acute trauma (within the first month after trauma because of unrepairable blind eye and fear of sympathetic ophthalmia), threatening or spontaneous perforation, cosmetic causes, and expulsive bleeding.

Statistical analysis was performed with Statistica 8.0 (StatSoft Inc., Tulsa, OK, USA). Data were expressed as median with standard deviation (SD). The chi-squared test was used to evaluate differences among groups. A *p* value lower than 0.05 was considered statistically significant.

## 3. Results

There were 266 (48.6%) males and 281 (51.4%) females in the study population. The patient age was 52.7 ± 24.5 years (range, 3 months to 100 years).

Age at the time of enucleation was as follows: for traumas, 48.6 ± 20.9 years (range, 3–100 years) (*n* = 92); for tumours, 51.2 ± 25.7 years (range, 0.3–87 years) (*n* = 259); for systemic diseases, 55.4 ± 23.4 years (range 1–81 years) (*n* = 28); for surgical diseases, 50.1 ± 27.2 years (range 0.25–94 years) (*n* = 86); for infectious or inflammatory diseases, 64.7 ± 18.9 years (range 15–93 years) (*n* = 63); for the miscellaneous group, 29.5 ± 18.2 years (range 5–64 years) (*n* = 11); and for the not-classified group, 58.3 ± 27.2 years (range 6–84 years) (*n* = 8). Within the tumour group, the age of patients at the time of surgery was significantly lower among retinoblastoma (RB) patients, 1.9 ± 1.5 years (range 0.3–8 years) (*n* = 36), than among those with uveal melanoma, 61.6 ± 13.9 years (range 18–87 years) (*n* = 200) (*p* < 0.0001).

**Figure 1:**
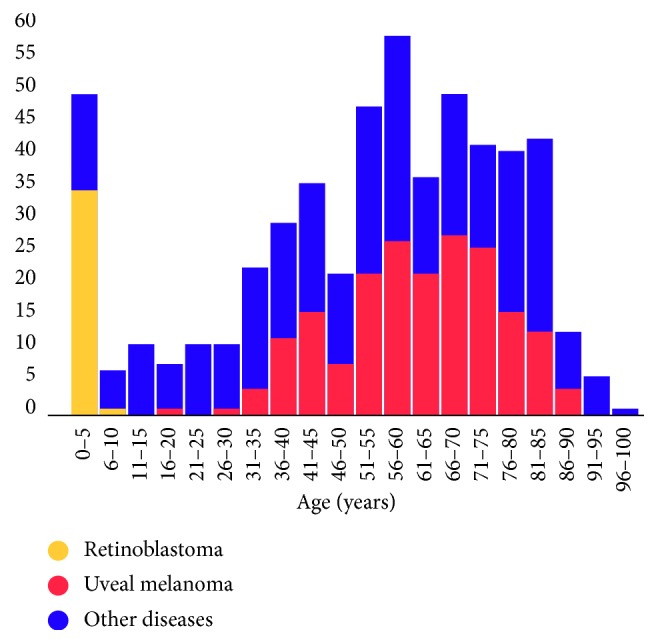
Age distribution of patients (each 5 years of age) at the time of enucleation with the primary enucleation indication of retinoblastoma, uveal melanoma, and other diseases, between January 2006 and December 2017 at the Department of Ophthalmology of Semmelweis University (Budapest, Hungary) (547 eyes of 543 patients).

The age distribution of the subjects (for each 5 years of age) with different primary enucleation indications is shown in Figure 1. Retinoblastoma was the most common primary enucleation indication in 0- to 5-year-old patients. Frequency of enucleations due to uveal melanoma increased from the 31 years of age in our sample (Figure 1).

**Figure 2:**
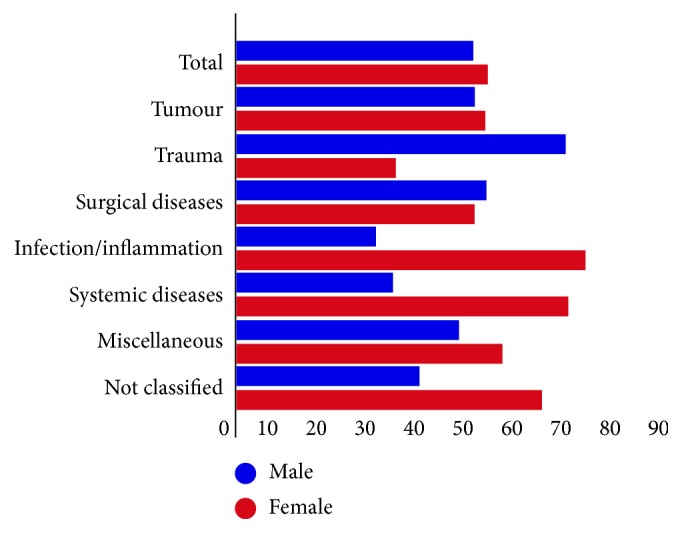
Sex distribution of patients who underwent enucleation (547 eyes of 543 patients) between January 2006 and December 2017 at the Department of Ophthalmology of Semmelweis University (Budapest, Hungary), distributed among primary enucleation indications. The numbers of patients in different groups were 259 for tumours, 92 for trauma, 86 for surgical diseases, 63 for infection or inflammation, 28 for systemic, 11 for miscellaneous diseases, and 8 not classified.

In the trauma group, the number of male patients was significantly higher than the number of female patients (*n* = 62/30, *p* < 0.0001); moreover, in the infection/inflammation group, the number of female subjects was significantly higher than the number of male subjects (*n* = 18/45, *p*=0.0007). There was no sex predominance in the systemic disease group (*n* = 9/19, *p*=0.0731), tumour group (*n* = 127/132, *p*=0.8570), or surgical disease group (*n* = 44/42, *p*=0.6085) (Figure 2). Because of small sample sizes, the miscellaneous and not classified groups were not analysed regarding age or gender distribution.

**Figure 3:**
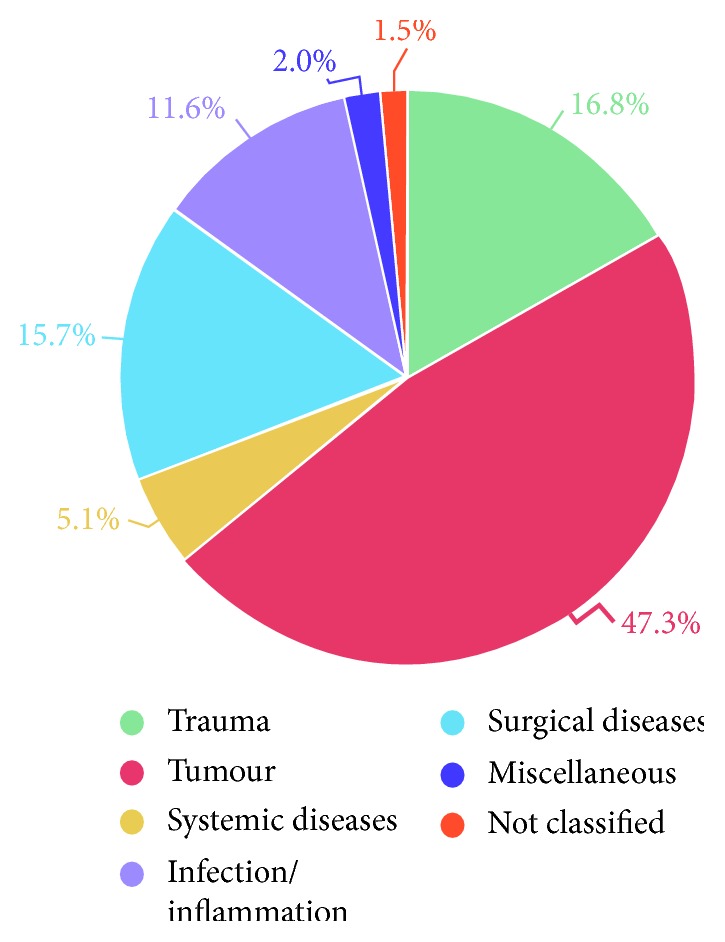
Percentages of primary enucleation indications between January 2006 and December 2017 at the Department of Ophthalmology of Semmelweis University (Budapest, Hungary).

**Figure 4:**
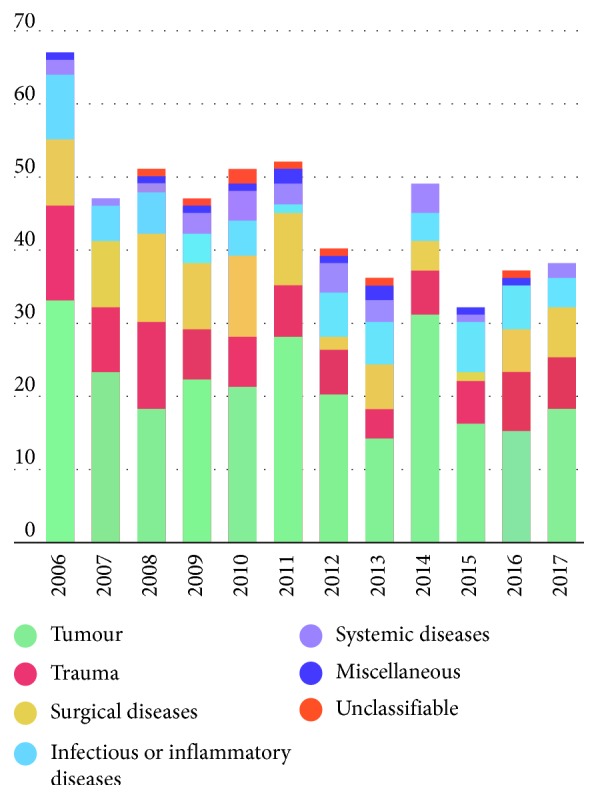
Primary enucleation indications between January 2006 and December 2017 at the Department of Ophthalmology of Semmelweis University (Budapest, Hungary).

*Primary enucleation indications* are displayed in Figures 3 and 4, as well as in Tables 1 and 2. These were (in decreasing order) tumours (*n* = 259; 47.3%) (intraocular, periocular, or intraorbital; Table 1), trauma (*n* = 92; 16.8%), surgical diseases (*n* = 86; 15.7%), infection or inflammation (*n* = 63; 11.6%), systemic diseases (*n* = 28; 5.1%, Table 2), miscellaneous diseases (*n* = 11; 2.0%), and not classified (*n* = 8; 1.5%). The number of enucleations per year showed a decreasing trend over the 12-year period (Figure 4).

Excluding eyes which were enucleated due to acute trauma, histopathologically there were 332 (64.2%) eyes with retinal detachment, 109 (21.1%) with angle closure, 105 (20.3%) with optic nerve head cupping, 88 (17.0%) with intraocular inflammatory reaction, 62 (12.0%) with subretinal, suprachoroidal, or vitreous haemorrhage, 52 (10.1%) with anterior chamber or pupillary membrane, 38 (7.4%) with intraocular ossification, and 21 (4.1%) with iris rubeosis.

In the tumour group, there was RB in 36 (13.9%) and malignant melanoma (MM) in 200 (77.2%) globes.

There were 29 (85.3%) undifferentiated and 5 (14.7%) differentiated RBs, and there were no histopathological data for 2 cases (incomplete documentation for this retrospective study). RBs involved the retinal pigment epithelium (*n* = 19, 55.9%), choroid (*n* = 16, 47.1%), vitreous body (*n* = 14, 41.2%), optic nerve head and optic nerve (with tumour-free margin in all cases) (*n* = 10, 29.4%), trabecular meshwork (*n* = 7, 20.6%), anterior or posterior chamber (*n* = 7, 20.6%), sclera (*n* = 1, 2.9%) and scleral canal (*n* = 1, 2.9%), or the orbit through the sclera (*n* = 1, 2.9%) and the orbit through the scleral canal (*n* = 1, 2.9%).

Choroidal MM was found in 163 (81.5%), ciliary body MM in 34 (17.0%), and iris MM in 3 (1.5%) eyes. Histopathologically, 108 (55.7%) were spindle-cell type, 32 (16.5%) were epithelioid-cell type, and 54 (27.8%) were mixed-cell-type MMs; there were no histopathological data available in 6 cases. Local tumour invasion was detected as follows: into scleral layers in 90 (46.4%), into the retina in 54 (27.8%), into the scleral canal in 19 (9.8%), into scleral emissary veins in 11 (5.7%), into the anterior chamber angle in 10 (5.2%), into the trabecular meshwork in 7 (3.6%), into Schlemm's canal in 6 (3.1%), and into the optic nerve (but with tumour-free margin in all cases) in 10 (5.2%) MM globes. Moreover, local invasion into the orbit was detected as follows: through scleral layers in 14 (7.2%), through the scleral canal in 7, (3.6%) and through scleral emissary veins in 4 (2.1%) eyes.

Other intraocular and periocular and intraorbital tumours (16; 6.2%) leading to enucleation are summarized in Table 1. Primary tumours in metastatic tumours of the uvea were lung adenocarcinoma (*n* = 2; 0.8%) and clear cell kidney carcinoma (*n* = 1; 0.4%).

Seven eyes (2.7%) were enucleated with clinical misdiagnosis of tumour. Three globes were enucleated with suspected RBs; among them, one had persistent hyperplastic primary vitreous (PHPV), one had Coats' disease, and one had retinal detachment due to incontinentia pigmenti, as their histological diagnoses. Four globes (1.5%) were enucleated with suspected uveal MM; among them, one (0.4%) had suprachoroidal haemorrhage, one (0.4%) had subretinal bleeding, one (0.4%) had old retinal detachment, and one (0.4%) had reactive gliosis of the retina, as their histological diagnoses.

In the trauma group, the primary location of the wound or injury was the cornea in 32 eyes (34.8%), corneoscleral tissue in 24 eyes (26.1%), sclera in 17 eyes (18.5%), at the optic nerve in 4 eyes (4.3%), and at the lens in 1 eye (1.1%) (blunt trauma). The place where the injury occurred was at home in 50 cases (54.3%), unknown in 18 (19.6%), in traffic in 8 (8.7%), at work in 7 (7.6%), in a violent act in 7 (7.6%), and in a sport accident in 2 (2.2%).

In the surgical disease group, 36 (41.9%) patients had glaucoma, 25 (29.1%) had retinal disease, 19 (22.1%) had lens-related disease, and 6 (7.0%) had corneal disease.

In the infection/inflammation group, 49 (77.8%) subjects had keratitis, 5 (7.9%) had chronic uveitis, 3 (4.8%) had iridocyclitis, 3 (4.8%) had chorioretinitis, 2 (3.2%) had scleritis, and 1 (1.6%) had endogenous endophthalmitis.

Systemic diseases that were enucleation indications are shown in Table 2.

In the miscellaneous diseases group, 5 (45.4%) patients had anterior staphyloma, 2 (18.2%) had persistent hyperplastic primary vitreous (PHPV), 1 (9.1%) had myopia, 1 (9.1%) had Rieger syndrome, 1 (9.1%) had facial nerve palsy, and 1 (9.1%) had microphthalmia.

*Immediate clinical enucleation indications* were (in decreasing order) tumours (*n* = 252, 46.1%), atrophia or phthisis bulbi (*n* = 101, 18.5%), infection or inflammation (*n* = 101, 18.5%), painful blind eye due to glaucoma (*n* = 61, 11.2%), acute trauma (*n* = 20, 3.7%), threatening or spontaneous perforation (*n* = 7, 1.3%), cosmetological reasons (*n* = 3, 0.5%), and expulsive bleeding (*n* = 2, 0.4%).

Among the 547 enucleated patients, 137 (25.0%) received a primary orbital implant at the time of eye removal. Types of implants were hydroxyapatite in 92 cases (67.2%), aluminium-oxide ceramic in 23 (16.8%), dermis-fat graft in 20 (14.6%), and silicone in 2 (1.5%).

## 4. Discussion

To our knowledge, this is the first study regarding ocular enucleations in Hungary and the first comprehensive study in Europe in the last 20 years. Enucleation is regarded as the last resort for many hopeless eye diseases, in which no other eye-preserving therapy is available; these include untreatable ocular malignancies, infections, inflammations, or painful blind eye. Due to differences in methodologies and definitions, comparisons between studies and results are not easy.

Regarding primary indications, patients with trauma were the youngest (48.6 years) and subjects with inflammatory or infectious diseases were the oldest (64.7 years) among enucleated persons. de Gottrau et al. [3] reported higher mean age (66.1 years) for surgical diseases, compared to our study (50.1 years); however, his mean ages for trauma (44.2 years) and inflammatory or infectious disease (67.6 years) groups were similar to those of our study (48.6 and 64.7 years).

The peak incidence of enucleation in Hungary (between 0–5 and 51–85 years) occurred in a similar age group among pediatric patients to that reported in China (between 0–10 and 31–40 years) [4] or in India (between 0 and 10 years) [6, 7] and in a similar age group among adult patients to that reported in Germany (between 61 and 75 years) [3]. In Hungary, in more than 82% of cases, enucleations were performed in subjects older than 30 years, while in India [6, 7] and Turkey [8], 82–84% and 54% of enucleations, respectively, were performed in subjects below 30 years of age.

In contrast to previous studies [6,7,9], our data showed a slight predominance of females undergoing enucleation, compared to males (51.4% vs. 48.6%). Trauma as a primary enucleation indication was more common among male than female patients (67.4% vs. 32.6%), similar to the findings of de Gottrau et al. (79% vs. 21%) [3], Freitag (75% vs. 25%) [10], and Cheng (81.3% vs. 18.7%) [4]. Furthermore, similar to de Gottrau et al. (62.5% vs. 37.5%) [3], ocular infections/inflammation (71.4% vs. 28.6%) were more common in females than in males.

In the present study, the most common *primary enucleation indications* were tumours (47.3%), trauma (16.8%), surgical diseases (15.7%) and infectious/inflammatory diseases (11.6%). In our study, there is an interesting inversion in the distribution of eyes with trauma and tumours, compared to previous studies. Previously, trauma was reportedly the most common primary enucleation indication (62.5% between 2003 and 2006 in China [4]; 37.4% between 1980 and 1990 in Germany [3]; 36.0% between 1982 and 2002 in Poland [11]), and tumours were the second most common indication (28.5% in China [4]; 20.7% in Poland [11]; 19.6% in Germany [3]). However, trauma is still considered the primary cause of enucleation in some developing countries [5]. This changing trend is well known [2, 9, 11–14], due to the improved management of eye traumas and better surgical tools, which help to prevent eye loss after severe ocular injuries. Therefore, trauma is not the most common indication for enucleation in developed countries anymore.

Furthermore, the number of enucleations with tumours did not decrease over time in the literature [9, 12]; a similar result was observed in our series (Figure 4) [15,16]. Similar to the findings of our study, tumours were also reported as the leading causes of enucleation in France [17], Turkey [8], and India [7] in 1996, 1997, and 2018, respectively.

In our study, the most common tumours were uveal MM among adults and RB in children, similar to the reports from China [4], Denmark [13], Germany [3], Iceland [9], Iran [18], and India [7]. The ratio of RB to MM was 1 : 5.5, which is similar to previous findings in our geographical region (France: 1 : 2 [17]; Poland: 1 : 13 [11]); notably, it contrasts with the ratios reported in Asia (China: 1.43 : 1 [4]; India: 7.3 : 1 [7]). It has been suggested that closer to Equator, MM has a lower prevalence, whereas RB has a higher prevalence [4]. Moreover, RB treatment methods are hardly available in developing countries [19].

In contrast to the results of studies from Western countries and similar to the findings from China [4], lung cancer was the most common primary tumour with ocular metastasis in enucleated globes. This is consistent with the fact that lung cancer is a leading cause of death in our country [20] because smoking is a serious public health problem in Hungary, compared to its frequency in other developed countries [21].

Clinical RB misdiagnosis (0.56%) was similar to the rate reported in China (0.48%) [4] and lower than that reported in India (6.0%) [6]. Clinical MM misdiagnosis (0.74%) was lower than the rate reported by de Gottrau et al. (2.3%) [3] and Cheng (4.0%) [4]. Additionally, no MMs were clinically misdiagnosed.

Sigurdsson et al. [9] found that work accidents were more common than those happening at home (34.7% vs. 26.4%). In contrast, we observed that home accidents (54.3%) were more common than work accidents (7.6%). The location of the primary wound in our series was similar to that reported by Cheng (mostly corneal/corneoscleral) [4], although strictly corneal injuries were slightly more common than corneoscleral wounds in our survey.

Systemic diseases were more common enucleation indications in the report by de Gottreau (17.1%) [3], than in our study (5.1%). Currently, through introduction of improved conservative and surgical treatment methods, we may prevent the most serious eye complications of systemic diseases. Diabetes mellitus (DM) is one of the most common causes of blindness and severe visual impairment in middle-aged people. The most dangerous and common complications of DM leading to enucleation are proliferative diabetic retinopathy and retinal vein occlusion [22, 23]. Enucleation due to systemic diseases has decreased remarkably in recent decades [12]. The proportions of retinal vein occlusion and diabetic retinopathy, as systemic disease causes of enucleation, were 57.7% and 32.7% in the study by de Gottrau et al. [3], whereas they were 14.3% and 17.9% in our study. Explanations are readily available: regular control and well-organized screening for DM, modern antidiabetic agents, introduction of panretinal photocoagulation, and vitreoretinal surgery can prevent and treat severe eye complications of DM [13].

Similar to the findings in the study by Cheng (5.3%) [4], we found iris rubeosis in 4.1% of the globes. This value is remarkably lower than that reported by de Gottrau et al. (48.0%) [3] and seems to refute the concept that most secondary angle closures follow rubeosis iridis [24].

The order of other primary enucleation indications in our study was similar to earlier reports. Tumour, trauma, and systemic diseases were followed by surgical (15.7% vs. 5.7–14.1%) and inflammatory/infectious diseases (11.6% vs. 1.7–7.0%) [3, 4]. Despite the development of microsurgical instruments and modern antibiotics, the frequency of surgical-related enucleations has not changed over time [3]. Anterior staphyloma (1.0%) was a rare primary enucleation indication, in contrast to the rate reported by Vemuganti from India [6] (49.0%), where anterior staphyloma was the most common primary cause of enucleation. Reportedly, anterior staphyloma is a rarity in Western countries but is a common condition in Asia [25].

The main *clinical immediate indications* for enucleation were tumours (46.1%), followed by atrophy or phthisis bulbi (18.5%) (similar to the new reports from India [7]), infection or inflammation (18.5%), painful blind eye secondary to glaucoma (11.2%), and acute trauma (3.7%). In China [4], 64.9% of all enucleations were performed in patients with atrophia or phthisis bulbi (36.4%) and tumours (28.5%), which is almost the same as our 64.6%, but in the reverse order. de Gottrau et al. [3] also reported similar results with one exception, almost 25 years ago. In his study, secondary glaucoma was the most common (34.9%) clinical indication for enucleation, whereas tumours were the second most common indication (21.7%). Most studies reported nearly similar proportions for glaucoma in enucleated eyes (USA: 8.0% [12]; China: 10.1% [4]; Denmark: 15.0% [13]; Turkey: 16.0% [8]; France: 16.0% [17]) to those found in our present work (11.2%). Worldwide, enucleation due to glaucoma has shown a decreasing trend over multiple decades. Setlur et al. found that, from the 1960s to the 2000s, there was a decreasing tendency, from 31% to 8.4% [12, 26]. The main explanation for this decrease may be introduction of modern therapeutic (antiglaucoma and antidiabetic agents) and surgical methods, which help to rescue many glaucomatous eyes and prevent serious eye complications [9, 13].

After excluding globes with tumour and acute trauma—similar to the method used by Cheng [4]—retinal detachment was most frequently associated with atrophia or phthisis bulbi (71.0%) in our series.

Approximately every fourth patient received an orbital implant following enucleation. The reason for this low proportion is that, in Hungary, financing of orbital implants is generally not covered by health insurance, nor is it included in the surgical costs of enucleation. Other studies have reported much higher rates of orbital implants (92–100%) [5, 27]. In the literature, hydroxyapatite implants were most frequently used (67.2%) [5, 18, 28].

## 5. Conclusions

In summary, intraocular tumours represent the most common clinicopathological indication for ocular enucleation in our study population. Following ocular trauma and systemic diseases, the rate of enucleation decreased in the last decade, compared to those previously reported in other developed countries. However, changes were not observed for surgical diseases, infectious and inflammatory causes, or miscellaneous and unclassified diseases. Orbital implant financing in Hungary should be increased to achieve better postoperative aesthetic rehabilitation following enucleation.

## Figures and Tables

**Table 1:** Primary enucleation indications of enucleated eyes (*n* = 259), in decreasing order, with suspected clinical diagnosis of tumour (uveal melanoma, retinoblastoma, other tumours, and false clinical suspicion of intraocular tumour) and histopathological diagnosis of the removed eyes with “other tumours.”

Primary enucleation indications	*n*	%
Uveal melanoma	200	77.2
Retinoblastoma	36	13.9
Other tumours	16	6.2
Basal-cell carcinoma	4	1.5
Ocular metastasis	3	1.2
Optic nerve glioma	2	0.8
Orbital adenoid cystic carcinoma	2	0.8
Ocular surface squamous neoplasia	2	0.8
Ocular lymphoma	1	0.4
Ocular multiple myeloma	1	0.4
Choroidal cavernous haemangioma	1	0.4
False clinical suspicion of intraocular tumour	7	2.7
Total	259	100

**Table 2:** Subspecification of systemic diseases as primary enucleation indication (*n* = 26), between January 2006 and December 2017, at the Department of Ophthalmology of Semmelweis University, Budapest, Hungary.

Clinicopathological diagnosis	*n*	%
Proliferative diabetic retinopathy	5	17.9
Rheumatoid arthritis	4	14.3
Retinal vein occlusion	4	14.3
Lyell's disease	3	10.7
Ocular ischaemic syndrome	2	7.1
Ocular cicatricial pemphigoid	2	7.1
Sjögren syndrome	2	7.1
Retinal artery occlusion	1	3.6
Sarcoidosis	1	3.6
Coats disease	1	3.6
Wegener's disease	1	3.6
von Hippel–Lindau disease	1	3.6
Marfan syndrome	1	3.6
Total	28	100

## Data Availability

The data used to support the findings of this study are included within the article.
